# *Levilactobacillus brevis* KU15147 Attenuates MIA-Induced Osteoarthritis by Modulating Inflammatory Responses and Cartilage Metabolism

**DOI:** 10.4014/jmb.2603.03042

**Published:** 2026-05-13

**Authors:** Na-Kyoung Lee, Yunjung Lee, Mijoo Choi, Eunju Park, Hyun-Dong Paik

**Affiliations:** 1 Department of Food Science and Biotechnology of Animal Resources, Konkuk University, Seoul 05029, Republic of Korea; 2 Department of Food and Nutrition, Kyungnam University, Changwon 51767, Republic of Korea; 3 View of Creativity, GHBio Co., Ltd., 120 Neungdong-Ro, Konkuk University, Seoul 05029, Republic of Korea

**Keywords:** *Levilactobacillus brevis* KU15147, Osteoarthritis, Monosodium iodoacetate, Inflammation, Cartilage metabolism

## Abstract

This study investigated the protective effects of *Levilactobacillus brevis* KU15147 on monosodium iodoacetate (MIA)-induced osteoarthritis in rats and inflammatory chondrocyte models. After 10 days of oral administration, osteoarthritis was induced via two intra-articular injections of MIA (50 μl of 60 mg/mL) at 3-day intervals. Experimental groups included normal control (NC), MIA-treated control (C), a positive control treated with indomethacin (PC, 3 mg/kg body weight/day), *L. brevis* KU15147 low-dose (15147-L, 1 × 10^8^ CFU/rat/day), and *L. brevis* KU15147 high-dose (15147-H, 1 × 10^9^ CFU/rat/day) groups. Knee thickness measurements, micro-computed tomography, and histological analyses were performed to evaluate joint structural changes. Serum levels of PGE_2_, LTB_4_, 5-lipoxgenase, tumor necrosis factor-α, interleukin (IL)-6, and IL-1β were measured, and cartilage metabolism- and inflammation-related gene expression were analyzed via quantitative real-time PCR. Furthermore, H_2_O_2_- or lipopolysaccharide (LPS)-stimulated chondrocytes were used to assess cytoprotective and anti-inflammatory effects *ex vivo*. MIA administration–induced cartilage degeneration, joint swelling, inflammatory mediator elevation, as well as catabolic and pro-inflammatory gene expression upregulation. *L. brevis* KU15147 treatment attenuated structural damage, suppressed systemic and local inflammatory responses, and restored the balance between anabolic and catabolic gene expression in both *in vivo* and *ex vivo* models. These findings suggest that *L. brevis* KU15147 exerts protective effects against osteoarthritis progression through the modulation of inflammatory pathways and cartilage metabolism.

## Introduction

Osteoarthritis (OA) is the most prevalent degenerative joint disease and a leading cause of disability worldwide [1, 2]. The disease is characterized by progressive cartilage degradation, subchondral bone remodeling, synovial inflammation, and chronic pain [3]. Although traditionally considered a “wear-and-tear” disorder, a growing body of evidence indicates that OA is driven by complex inflammatory and metabolic mechanisms involving chondrocytes, immune mediators, and oxidative stress [4, 5].

Chondrocytes are the sole resident cells within articular cartilage and are responsible for maintaining extracellular matrix (ECM) homeostasis [6]. The ECM is primarily composed of type II collagen and aggrecan, which provide tensile strength and compressive resistance, respectively [7]. Under pathological conditions, pro-inflammatory cytokines such as tumor necrosis factor-α (TNF-α) and interleukin-1β (IL-1β) stimulate the production of matrix metalloproteinases (MMPs), leading to cartilage degradation [8, 9]. Among these enzymes, MMP-3, -7, and -13 play critical roles in ECM breakdown [10]. Tissue inhibitors of metalloproteinases (TIMPs), including TIMP-1 and -3, regulate MMP activity; notably, any disruption of the MMP/TIMP balance accelerates cartilage destruction [11].

Oxidative stress is another major contributor to OA pathogenesis. Reactive oxygen species (ROS) impair chondrocyte viability, suppress anabolic gene expression, and upregulate catabolic signaling pathways [12]. Hydrogen peroxide (H_2_O_2_) is widely used to mimic oxidative stress-induced cartilage damage *in vitro* [13]. Additionally, lipopolysaccharide (LPS) stimulation induces inflammatory responses in chondrocytes via the activation of NF-κB signaling and its downstream mediators [14]. These experimental models provide robust platforms for evaluating potential therapeutic agents targeting OA-related inflammation and cartilage metabolism.

Monosodium iodoacetate (MIA) irreversibly inhibits glyceraldehyde-3-phosphate dehydrogenase (GAPDH), disrupting glycolysis in chondrocytes [15]. This metabolic blockade leads to energy depletion, accumulation of reactive oxygen species (ROS), and activation of stress and inflammatory responses. These events promote chondrocyte apoptosis, upregulation of catabolic mediators (*e.g.*, MMPs, COX-2, and 5-LO), and progressive cartilage degradation, thereby producing a clinically relevant OA phenotype in the MIA model [15, 16].

Current pharmacological treatments for OA, including nonsteroidal anti-inflammatory drugs (NSAIDs) such as indomethacin, primarily alleviate symptoms but fail to prevent cartilage degeneration effectively [17]. Furthermore, long-term NSAID use is also associated with gastrointestinal and cardiovascular adverse effects [18]. Therefore, developing safer and more effective therapeutic strategies remains a priority.

Recent studies have highlighted the potential role of probiotics in modulating inflammatory and metabolic disorders [19]. Probiotic strains, particularly those belonging to the genus *Lactobacillus*, elicit anti-inflammatory and antioxidant effects by regulating cytokine production and immune responses [20]. Emerging evidence suggests that the gut–joint axis may influence OA progression; specifically, modulation of the intestinal microbiota attenuates joint inflammation and cartilage degradation [21]. Several experimental studies have demonstrated that probiotic supplementation reduces inflammatory mediators and preserves cartilage integrity in OA models [22].

*Levilactobacillus brevis* KU15147 has demonstrated anti-inflammatory effects via suppressing NF-κB, AP-1, and MAPK signaling pathways [23]. However, its protective effects against oxidative stress- and inflammation-mediated cartilage damage remain to be fully elucidated. Therefore, the present study evaluated the anti-osteoarthritic effects of *L. brevis* KU15147 using *in vivo* and *ex vivo* experimental models. We investigated its effects on inflammatory mediators, cartilage metabolism-related gene expression, and extracellular matrix homeostasis in monosodium iodoacetate (MIA)-induced OA rats, as well as in H_2_O_2_- and LPS-stimulated chondrocytes.

## Materials and Methods

### Sample Preparation

*Levilactobacillus brevis* KU15147 was cultured in De Man, Rogosa, and Sharpe (MRS) broth (Difco, USA) at 37°C for 24 h under anaerobic conditions. The bacterial cells were harvested by centrifugation at 5,000 × *g* for 10 min, washed twice with sterile phosphate-buffered saline (PBS), and lyophilized. Viable cell counts were determined using serial dilution and plate counting on MRS agar. For animal administration and *ex vivo* experiments, the bacterial suspension was freshly prepared in sterile saline at the indicated concentrations.

### Experimental Design

Male Sprague–Dawley rats (7–8 weeks old) were obtained from an accredited laboratory animal supplier and housed under standard conditions (22 ± 2°C, 55 ± 5% humidity, and a 12 h light/dark cycle) with *ad libitum* access to food and water.

OA was induced by intra-articular injection of MIA into the right knee joint. Animals were randomly assigned to the following groups: normal control (NC); MIA-induced OA (C); MIA + indomethacin (3 mg/kg body weight (BW)/day, PC); MIA + *L. brevis* KU15147 low-dose (1 × 10^8^ CFU/rat/day, 15147-L); and MIA + *L. brevis* KU15147 high-dose (1 × 10^9^ CFU/rat/day, 15147-H). *L. brevis* KU15147 or indomethacin was orally administered once daily throughout the experimental period. After 10 days of oral administration, two intra-articular injections (50 μL of 60 mg/mL) were administered at 3-day intervals.

All animal procedures were approved by the Institutional Animal Care Committee of Kyungnam University (KUICA-22-07) and followed established guidelines.

### Histological Observation of Articular Cartilage

Histological changes were assessed to determine the effect of *L. brevis* KU15147 treatment on the knee joint. The animals were perfused via the ascending aorta using 10% neutral buffered formalin (pH 7.4). The knee joints, including the patella and joint capsule, were resected, maintained in the same fixative for an additional 48 h at 4°C, and then embedded in paraffin. The tissue sections were stained with hematoxylin and eosin (H&E) to evaluate the proteoglycan content.

### Measurement of Knee Joint Swelling and Micro-CT Analysis

Knee joint diameters were measured using a digital caliper at predetermined time points. Bone mineral density (BMD) and structural changes of the knee joint were evaluated using micro-computed tomography (micro-CT). Micro-CT imaging was performed using a Viva CT 80 system (Scanco Medical AG, Switzerland). Three-dimensional (3D) reconstruction and quantitative analysis were performed according to the manufacturer’s instructions.

### Serum Cytokine Analysis

At the end of the experimental period, blood samples were collected and centrifuged at 3,000 × *g* for 15 min to isolate serum. Levels of TNF-α, IL-6, IL-1β, PGE_2_, and LTB_4_ were measured using commercial enzyme-linked immunosorbent assay (ELISA) kits according to the manufacturer’s protocols (R&D Systems, USA).

### H_2_O_2_/LPS-Induced Oxidative Stress Model and Treatment

Primary rat chondrocytes were cultured in Dulbecco’s Modified Eagle Medium (DMEM; Gibco, USA) supplemented with 10% fetal bovine serum (FBS) and 1% penicillin–streptomycin at 37°C in a humidified atmosphere containing 5% CO_2_.

Oxidative stress-induced inflammatory conditions were established by co-treating cells with hydrogen peroxide (H_2_O_2_; 600 μM) or LPS (50 μg/ml) and *L. brevis* KU15147 at concentrations ranging from 5 × 10^7^ to 5 × 10^10^ CFU for 24 h. Indomethacin (100 μM) was used as a positive control.

### RNA Isolation and Quantitative Real-Time PCR

Total RNA was extracted using a commercial RNA isolation kit. Complementary DNA (cDNA) was synthesized from 1 μg of total RNA using a reverse transcription kit. Quantitative real-time PCR (qPCR) was performed using SYBR Green Master Mix on a real-time PCR system.

Expression levels of genes encoding TNF-α, IL-6, IL-1β, iNOS, COX-2, aggrecan, collagen types I, II, and X, MMP-3, -7, and -13, as well as TIMP-1 and -3 were normalized to that of the gene encoding GAPDH. The primer sequences used in this study are listed in Table 1. Relative gene expression levels were calculated using the 2^-ΔΔCt^ method.

### Statistical Analyses

All data are presented as the mean ± standard deviation (SD). Statistical significance was determined using one-way analysis of variance (ANOVA) followed by Tukey’s multiple comparison test. Differences were considered statistically significant at *p* < 0.05.

## Results

### Effects of *L. brevis* KU15147 on Organ Weights

Relative organ weights were evaluated to assess potential systemic effects (Table 2). The C group showed a significant increase in heart, liver, and spleen weights compared with the NC group. Notably, liver weight was significantly increased in the 15147-L group; however, this increase was not observed in the 15147-H group, which remained comparable to the NC group. No significant differences were observed in kidney weights among the experimental groups. These results suggest that *L. brevis* KU15147 did not induce marked organ toxicity under the specified experimental conditions.

### Histological Observation and Micro-CT Analysis of Articular Cartilage

**Fig. 1:**
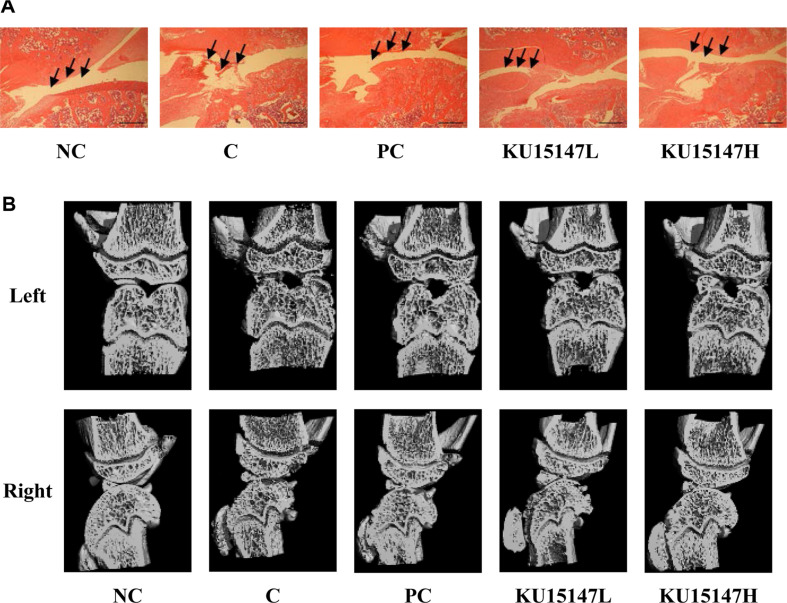
Histological and bone mineral density (BMD) analysis of the knee joint in rats with MIA-induced osteoarthritis. (**A**) Histological observation of knee joint tissues using hematoxylin and eosin (H&E) staining. (**B**) Changes in BMD of the knee joint measured via micro-computed tomography (micro-CT). NC: treatment without monosodium iodoacetate (MIA) injection; C: treatment with MIA injection; PC: treatment with MIA + indomethacin (3 mg/kg body weight/day); 15147-L: treatment with MIA + *L. brevis* KU15147 low-dose (1 × 10^8^ CFU/rat/day); 15147-H: treatment with MIA + *L. brevis* KU15147 (1 × 10^9^ CFU/rat/day). The scale bars, represented as “-”, indicate a physical distance of 100 μm.

Histopathological examination of the knee joint sections was performed to evaluate structural changes in the articular cartilage (Fig. 1A). The NC group exhibited intact articular cartilage characterized by a smooth surface and well-preserved cartilage structure. Conversely, the C group exhibited severe cartilage erosion, surface irregularity, and marked structural disruption, consistent with osteoarthritic alterations.[Table T3]

Treatment with *L. brevis* KU15147 attenuated these histopathological changes. The 15147-L group showed partial preservation of cartilage structure with reduced surface damage compared with the C group. The 15147-H group demonstrated markedly improved cartilage integrity, with decreased erosion and inflammatory infiltration. Overall, *L. brevis* KU15147 treatment reduced MIA-induced histological damage in a dose-dependent manner.

Structural changes in the knee joint were evaluated using micro-CT (Fig. 1B and Table 4). Compared with the NC group, the C group exhibited reduced trabecular density and a disrupted bone microarchitecture. Quantitative analysis revealed that BMD were significantly decreased in the PC group compared to the C group (*p* < 0.05). Furthermore, trabecular number (Tb.N) were reduced, whereas trabecular separation (Tb.Sp) was increased relative to the NC group (*p* < 0.05). Treatment with KU15147 improved bone structural parameters compared with the C group (Table 4). In particular, BMD in the 15147-H group was restored to a level comparable to that of the C group. Although BV/TV and Tb.Th were not fully restored to normal levels, partial improvements were observed. Moreover, Tb.Sp was increased in the *L. brevis* KU15147-treated groups compared with the PC group (*p* < 0.05). These results indicate that *L. brevis* KU15147 attenuated MIA-induced subchondral bone loss.

### Knee Joint Swelling Assessment

Knee thickness was measured at 7, 10, and 13 days after the second intra-articular injection of monosodium iodoacetate (MIA) administered at 3-day intervals (Table 4). In both the left and right knees, the C group showed a significant increase in knee thickness compared with the NC group at all time points, indicating the successful induction of joint inflammation.

On day 7, the NC group displayed a knee joint thickness of 20.0 ± 0.3 mm, whereas the joints of the C group exhibited a significantly greater thickness of 21.4 ± 0.2 mm (*p* < 0.05). The 15147-L and 15147-H groups showed comparable values of 20.5 ± 0.2 mm and 20.4 ± 0.2 mm, respectively. The joints of the PC group showed a value of 20.2 ± 0.2 mm. On day 10, the joints of the C group remained significantly thicker (18.8 ± 0.4 mm) compared to those of the NC group (17.3 ± 0.2 mm) and both 15147-L (17.8 ± 0.2 mm) and PC (18.1 ± 0.2 mm) groups. The joints of the 15147-H group showed no significant difference at 18.0 ± 0.3 mm. By day 13, the joints of the C group maintained the maximal thickness at 18.8 ± 0.5 mm. The NC group exhibited a baseline thickness of 17.2 ± 0.2 mm. The joints of the 15147-L and 15147-H groups had consistent values of 17.8 ± 0.2 mm and 18.0 ± 0.2 mm, whereas the joints of the PC group reported 17.8 ± 0.2 mm with a significant difference noted compared to those of the C group, respectively. Statistical analysis indicated significant differences among the groups, particularly the joints of the C group, which exhibited the most pronounced swelling throughout the assessment period (*p* < 0.05).

### Serum Levels of PGE_2_, Leukotriene B_4_, 5-Lipoxygenase, and Pro-Inflammatory Cytokines

**Fig. 2:**
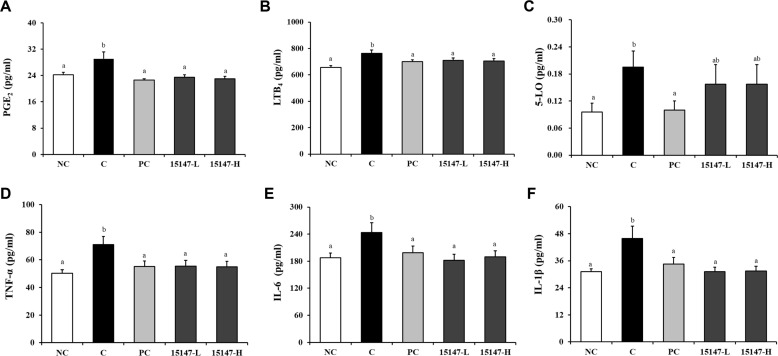
Effects of *Levilactobacillus brevis* KU15147 on serum inflammatory mediators in rats with MIA-induced osteoarthritis, as measured by ELISA. (**A**) PGE_2_, (**B**) LTB_4_, (**C**) 5-LO, (**D**) TNF-α, (**E**) IL-6, and (**F**) IL-1β. NC: treatment without monosodium iodoacetate (MIA) injection; C: treatment with MIA injection; PC: treatment with MIA + indomethacin (3 mg/kg body weight/day); 15147-L: treatment with MIA + *L. brevis* KU15147 low-dose (1 × 10^8^ CFU/rat/day); 15147-H: treatment with MIA + *L. brevis* KU15147 (1 × 10^9^ CFU/rat/day). Data are presented as the mean ± standard deviation (SD), and different letters on error bars indicate significant differences (*p* < 0.05).

Changes in inflammatory mediators associated with cartilage degradation are presented in Fig. 2. The MIA-induced C group exhibited significantly elevated levels of PGE_2_, LTB_4_, and 5-LO compared with the NC group. Administration of the PC and 15147-treated groups significantly reduced serum PGE_2_ and LTB_4_ levels compared with the C group. Although 5-LO levels were not significantly different among the treatment groups, a downward trend was observed after *L. brevis* KU15147 administration. Similarly, serum concentrations of TNF-α, IL-6, and IL-1β were significantly increased in the C group compared with those in the N group. Treatment with PC and all *L. brevis* KU15147 doses significantly suppressed TNF-α, IL-6, and IL-1β levels relative to those of the PC group, indicating the attenuation of systemic inflammatory responses.

The marked elevation of eicosanoid mediators (PGE_2_ and LTB_4_) in the PC group suggests the activation of COX- and LOX-dependent inflammatory pathways after MIA induction. *L. brevis* KU15147 administration effectively suppressed these mediators, supporting its potential role in modulating arachidonic acid metabolism. Furthermore, the significant reduction in circulating TNF-α, IL-6, and IL-1β levels indicates that *L. brevis* KU15147 mitigates the systemic inflammation associated with OA progression.

### Gene Expression of Cartilage-Related Markers in Joint Tissues

**Fig. 3:**
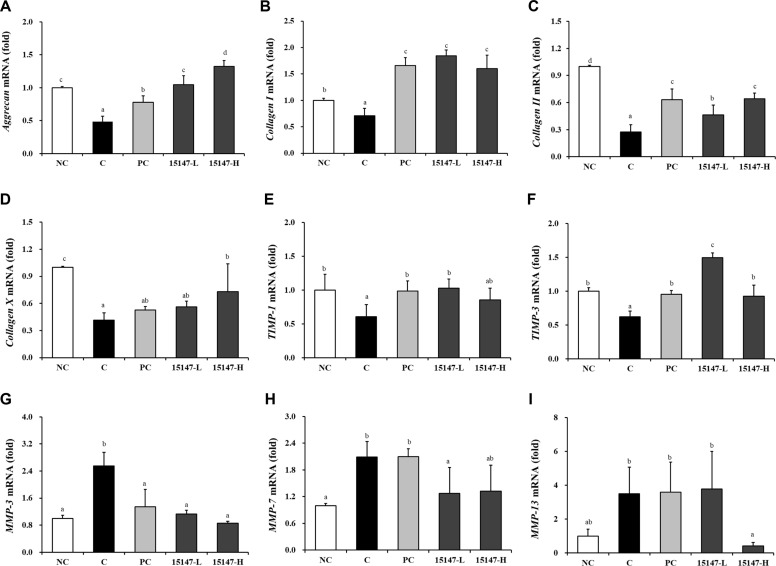
Effects of *Levilactobacillus brevis* KU15147 on cartilage metabolism-related gene expression in joint tissues of rats with MIA-induced osteoarthritis, as measured by quantitative real-time PCR. (**A**) *Aggrecan*, (**B**) *Collagen type I*, (**C**) *Collagen type II*, (**D**) *Collagen type X*, (**E**) *TIMP-1*, (**F**) *TIMP-3*, (**G**) *MMP-3*, (**H**) *MMP-7*, and (**I**) *MMP-13*. NC: treatment without monosodium iodoacetate (MIA) injection; C: treatment with MIA injection; PC: treatment with MIA + indomethacin (3 mg/kg body weigh/day); 15147-L: treatment with MIA + *L. brevis* KU15147 low-dose (1 × 10^8^ CFU/rat/day); 15147-H, treatment with MIA + *L. brevis* KU15147 (1 × 10^9^ CFU/rat/day). Data are presented as the mean ± standard deviation (SD), and different letters on error bars indicate significant differences (*p* < 0.05).

The mRNA expression of anabolic and catabolic genes associated with cartilage metabolism was analyzed in joint tissues (Fig. 3). Compared with the NC group, the osteoarthritis-induced C group showed significant downregulation of anabolic markers, including the genes encoding aggrecan, collagen types I, II, and X, as well as TIMP-1 and -3 (*p* < 0.05).

Administration of *L. brevis* KU15147 significantly and dose-dependently increased the expression of genes encoding aggrecan, collagen types I, II, and X compared with the C group (*p* < 0.05). The expression levels of *TIMP-1* and *-3* were also significantly elevated in the 15147-treated groups, with greater upregulation observed in the 15147-L group than that in the 15147-H group.

Conversely, the expression of the genes encoding the catabolic markers MMP-3, -7, and -13 was significantly increased in the PC group compared with the NC group (*p* < 0.05). Treatment with KU15147 significantly reduced *MMP-3* expression in all treated groups relative to the PC group (*p* < 0.05). Furthermore, MMP-7 and -13 expression levels were significantly reduced in the 15147-L and 15147-H groups, respectively.

### Gene Expression Analysis of Inflammation-Related Markers in Joint Tissues

**Fig. 4:**
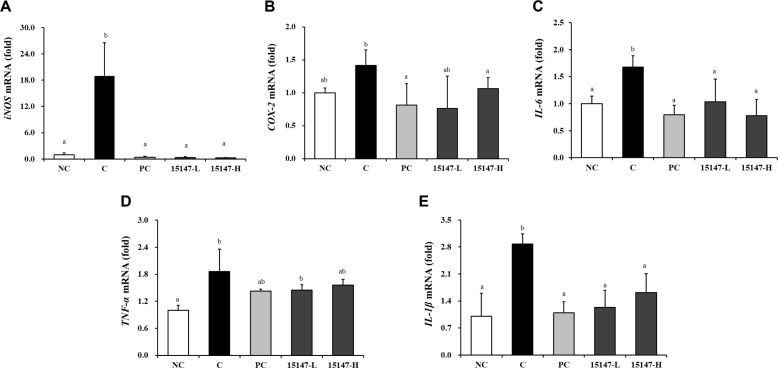
Effects of *Levilactobacillus brevis* KU15147 on inflammatory gene expression in joint tissues of rats with MIA-induced osteoarthritis, as measured via quantitative real-time PCR. (**A**) *iNOS*, (**B**) *COX-2*, (**C**) *TNF-α*, (**D**) *IL-6*, and (**E**) *IL-1β*. NC: treatment without monosodium iodoacetate (MIA) injection; C: treatment with MIA injection; PC: treatment with MIA + indomethacin (3 mg/kg body weight/day); 15147-L: treatment with MIA + *L. brevis* KU15147 low-dose (1 × 10^8^ CFU/rat/day); 15147-H: treatment with MIA + *L. brevis* KU15147 (1 × 10^9^ CFU/rat/day). Data are presented as the mean ± standard deviation (SD), and different letters on error bars indicate significant differences (*p* < 0.05).

The mRNA expression levels of inflammation-related genes in articular cartilage are presented in Fig. 4. Compared with the NC group, the osteoarthritis-induced C group exhibited significantly increased expression of the genes encoding iNOS, IL-6, and IL-1β (*p* < 0.05). All *L. brevis* KU15147-treated groups exhibited a significant reduction in *iNOS*, *IL-6*, and *IL-1β* expression compared with the C group (*p* < 0.05). *COX-2* expression did not differ significantly between the NC and C groups. However, the 15147-H group demonstrated a significant decrease in *COX-2* expression compared with the C group. *TNF-α* mRNA expression was not significantly altered by *L. brevis* KU15147 treatment under these experimental conditions.

### Ex Vivo Analysis of the Anti-Osteoarthritic Effects of *L. brevis* KU15147

To evaluate the cytoprotective effects of *L. brevis* KU15147 under oxidative stress, primary chondrocytes were treated with 600 μM H_2_O_2_. This treatment significantly reduced cell viability to 58.39 ± 1.12% compared with that of the untreated control group (Fig. S1A). Treatment with KU15147 (5 × 10^7^ – 5 × 10^10^ CFU) dose-dependently restored cell viability to 75.91 ± 4.01%, 78.89 ± 5.08%, 79.17 ± 1.17%, and 99.16 ± 0.65%, respectively. Notably, the highest concentration of *L. brevis* KU15147 almost entirely reversed the H_2_O_2_-induced cytotoxicity, reaching a level comparable to that of the untreated control. Indomethacin (100 μM), used as the positive control, restored cell viability to 100.29 ± 1.80%, confirming the validity of the experimental model.

**Fig. 5:**
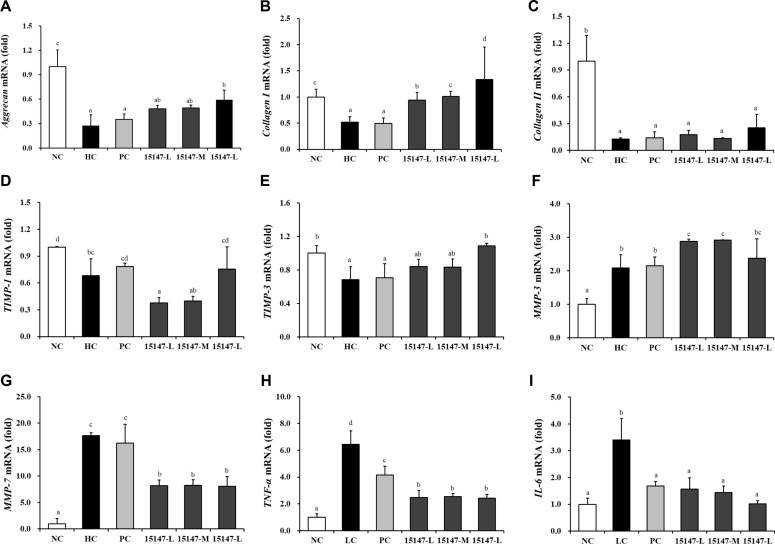
Effects of *Levilactobacillus brevis* KU15147 on cartilage metabolism-related gene expression in inflamed chondrocytes. (**A–G**) Impact of *L. brevis* KU15147 on cartilage metabolism-related gene expression in H_2_O_2_-stimulated chondrocytes: (**A**) *aggrecan*, (**B**) *collagen type I*, (**C**) *collagen type II*, (**D**) *TIMP-1*, (**E**) *TIMP-3*, (**F**) *MMP-3*, and (**G**) *MMP-7*. (H–I) Effects of *L. brevis* KU15147 on inflammatory cytokine gene expression in LPS-stimulated chondrocytes: (**H**) *TNF-α* and (**I**) *IL-6.* Gene expression levels were normalized to that of *GAPDH* and calculated using the 2^-ΔΔCt^ method. NC: treatment without H_2_O_2_ or LPS; HC or LC: treatment with H_2_O_2_ (600 μM) or LPS (50 μg/ml); PC: treatment with H_2_O_2_ (600 μM) or LPS (50 μg/ml) + indomethacin (100 μM), 15147-L, M, and H: treatment with H_2_O_2_ (600 μM) or LPS (50 μg/ml) + (5 × 10^7^, 5 × 10^8^, and 5 × 10^9^ CFU, respectively). Data are presented as the mean ± standard deviation (SD), with different letters indicating statistically significant differences (*p* < 0.05).

H_2_O_2_ treatment significantly decreased the expression of the genes encoding aggrecan, collagen types I and II, as well as TIMP-1 and -3, compared to that of the untreated control group (*p* < 0.05) (Fig. 5A–5G). Treatment with KU15147 significantly restored the expression of these genes in a dose-dependent manner relative to that of the H_2_O_2_-treated group (HC) (*p* < 0.05). H_2_O_2_ exposure significantly increased the expression of *MMP-7* compared to that of the negative control (*p* < 0.05). However, MMP-3 did not show a significant reduction compared to the H_2_O_2_-treated group (HC) (*p* > 0.05).

Primary chondrocytes were exposed to 50 μg/ml LPS to induce cytotoxicity, and cell viability was assessed using the MTT assay (Fig. S1B). LPS treatment significantly decreased cell viability to 73.75 ± 3.50% compared with that of the untreated control group (*p* < 0.05). Treatment with *L. brevis* KU15147 (5 × 10^7^ – 1 × 10^9^ CFU) significantly increased cell viability compared with that of the LPS group (*p* < 0.05), restoring viability to levels ranging from 99.46% to 104.60%. Indomethacin (100 μM) also significantly improved cell viability to 92.90 ± 4.96% as compared with that of the LPS group (*p* < 0.05).

LPS stimulation significantly increased *TNF-α* and *IL-6* gene expression compared with that of the untreated control group (*p* < 0.05) (Fig. 5H–I). All *L. brevis* KU15147-treated groups reduced LPS-induced *TNF-α* and *IL-6* gene expression (*p* < 0.05). The reduction in TNF-α expression observed in the *L. brevis* KU15147-treated groups was greater than that in the indomethacin-treated group (*p* < 0.05).

## Discussion

OA is hallmarked by progressive cartilage degradation, subchondral bone remodeling, synovial inflammation, and chronic pain. A growing body of evidence indicates that inflammatory mediators, oxidative stress, and the dysregulation of ECM turnover play central roles in OA pathogenesis [1, 2, 5]. Herein, *L. brevis* KU15147 significantly ameliorated MIA-induced OA in rats by reducing joint swelling, improving BMD, suppressing systemic inflammatory mediators, and restoring cartilage metabolic balance. *Ex vivo* experiments further demonstrated cytoprotective and anti-inflammatory effects on H_2_O_2_- and LPS-stimulated primary chondrocytes. *L. brevis* KU15147 may exert protection in the MIA model — (1) modulation of systemic and local inflammation via dampening of proinflammatory cytokines and eicosanoids (PGE_2_ and LTB_4_), (2) reduction of oxidative stress through antioxidant activities or induction of host antioxidant responses, and (3) indirect modulation of cartilage catabolic signaling (NF-κB and MAPKs) that are activated downstream of metabolic injury. We also cite studies where other *Lactobacillus* strains altered oxidative stress markers and MAPK/NF-κB pathways in joint or inflammatory models.

Previous studies have reported that *L. brevis* KU15147 exerts anti-inflammatory and antioxidant effects in LPS-stimulated RAW 264.7 macrophages [23]. The KU15147 strain reduced NO and PGE_2_ production through the downregulation of *iNOS* and *COX-2* expression without inducing cytotoxicity. In addition, the production of TNF-α, IL-1β, and IL-6 was significantly attenuated via the NF-κB, AP-1, and MAPK signaling pathways, accompanied by reduced intracellular ROS generation. The present *in vivo* findings are consistent with these observations and suggest that the modulation of these inflammatory signaling pathways may underlie the protective effects of *L. brevis* KU15147 in OA.

Firstly, organ weight changes (*e.g.*, systemic inflammation causing splenomegaly, hepatic changes due to inflammatory mediators or metabolic shifts) were confirmed. We clarify whether the observed organ weight differences reached statistical significance and whether they correlated with inflammatory markers. However, we will perform spleen histology and liver ALT/AST for further study.

The MIA-induced OA is widely used because it induces chondrocyte apoptosis, cartilage erosion, and subchondral bone alterations resembling human OA pathology. Elevated levels of TNF-α, IL-1β, and IL-6 induce MMP production, accelerate ECM degradation, and inhibit collagen synthesis [5, 24]. In this study, *L. brevis* KU15147 significantly reduced serum TNF-α, IL-6, and IL-1β levels in MIA-induced OA rats, consistent with previous reports indicating that probiotic strains modulate NF-κB-dependent inflammatory responses [19, 25, 26]. Furthermore, the suppression of PGE_2_ and LTB_4_ suggests the inhibition of arachidonic acid-related inflammatory pathways, which are critically involved in OA progression [27, 28].

Cartilage destruction in OA results from an imbalance between anabolic mediators (*e.g.*, aggrecan and collagen type II) and catabolic enzymes (*e.g.*, MMP-3 and -13) [1, 29]. MMP-13 plays a pivotal role in collagen type II degradation and is strongly associated with OA severity [10]. *L. brevis* KU15147 upregulated aggrecan and collagen-related genes while downregulating the expression of the genes encoding MMP-3, -7, and -13, indicating the restoration of ECM homeostasis. Additionally, the modulation of TIMP-1 and -3 suggests the reestablishment of the MMP/TIMP regulatory axis for cartilage preservation [30]. These findings support the notion that *L. brevis* KU15147 protects cartilage via rebalancing anabolic–catabolic signaling.

Subchondral bone remodeling is another hallmark of OA progression. Micro-CT analysis demonstrated that *L. brevis* KU15147 improved BMD and attenuated cartilage erosion. Since altered bone–cartilage crosstalk contributes to OA development, the stabilization of subchondral bone may partially account for the observed protective effects [31, 32].

Oxidative stress is a major contributor to chondrocyte apoptosis and ECM degradation [32]. In the *ex vivo* model, *L. brevis* KU15147 dose-dependently restored cell viability in H_2_O_2_-stimulated chondrocytes and partially normalized anabolic gene expression. Because ROS activate the MAPK and NF-κB pathways, leading to increased MMP production and inflammatory cytokine release [12, 33], the attenuation of oxidative stress may complement the anti-inflammatory activity of *L. brevis* KU15147.

The gut–joint axis has emerged as a critical factor in OA pathogenesis. Gut dysbiosis and elevated circulating LPS levels promote systemic, low-grade inflammation and joint degeneration [21, 34, 35]. Probiotic supplementation has been shown to reduce inflammatory markers and improve symptoms in both experimental and clinical OA settings [22, 25, 27]. In the present study, *L. brevis* KU15147 suppressed LPS-induced TNF-α and IL-6 expression in primary chondrocytes, suggesting the modulation of upstream inflammatory signaling rather than the sole inhibition of cyclooxygenase activity. To specify gut-join axis, we will perform in further study.

Collectively, these findings demonstrate that *L. brevis* KU15147 alleviates inflammation, suppresses catabolic signaling, and modulates cartilage matrix metabolism in MIA-induced OA. Although further studies are required to elucidate the precise molecular mechanisms and clinical relevance, *L. brevis* KU15147 may represent a promising probiotic candidate for the management of osteoarthritis.

## Conclusion

*L. brevis* KU15147 significantly attenuated MIA-induced osteoarthritis *in vivo* and exerted protective effects in oxidative stress- and LPS-induced inflammatory models *ex vivo*. The KU15147 strain reduced knee joint swelling, improved BMD, and suppressed systemic inflammatory mediators, including PGE_2_, LTB_4_, TNF-α, IL-6, and IL-1β. Furthermore, *L. brevis* KU15147 restored cartilage metabolic balance by upregulating anabolic markers (aggrecan and collagen-related genes) while downregulating catabolic enzymes (MMP-3, -7, and -13), thereby reestablishing the MMP/TIMP regulatory axis. *Ex vivo* analyses further demonstrated that *L. brevis* KU15147 protected primary chondrocytes against H_2_O_2_- and LPS-induced cytotoxicity and significantly suppressed pro-inflammatory gene expression, particularly those encoding TNF-α and IL-6. These findings suggest that *L. brevis* KU15147 mitigates osteoarthritic progression through the coordinated modulation of inflammatory signaling and extracellular matrix homeostasis. Collectively, our results provide mechanistic evidence supporting *L. brevis* KU15147 as a promising probiotic candidate for the prevention or management of osteoarthritis. Future studies involving microbiome profiling, signaling pathway validation, and clinical trials are warranted to further elucidate its therapeutic potential.

## Figures and Tables

**Table 1:** Primer sequences used for quantitative real-time PCR analysis.

Gene	Forward primer (5′→3′)	Reverse primer (5′ → 3′)
*Aggrecan*	5′- CTTCAGGTGTGCCTTTTGCC-3′	5′- GGCTGTTTCTGCTGTTTGGG-3′
*Collagen type I*	5′-AGCTGCATACACAATGGCCT-3′	5′- CTTGGGTCCCTCGACTCCTA-3′
*Collagen type II*	5′-GAGCAGCAAGAGCAAGGAGA-3′	5′-TGAGCAAGGCCTTCTTGAGG-3′
*Collagen type X*	5′-TGATCCTGGAGTGGGAGGAG-3′	5′- GCCCATTGAGGCCCTTAGTT-3′
*TIMP-1*	5′-GCCTCTGGCATCCTCTTGTT-3′	5′- AGCGTCGAATCCTTTGAGCA-3′
*TIMP-3*	5′- GGCAAGATGTACACAGGGCT-3′	5′- TGATAGCCAGGGTACCCGAA-3′
*MMP-3*	5′- CCCTCTATGGACCTCCCACA-3′	5′-GGATGGAAGAGACGGCCAAA-3′
*MMP-7*	5′- TGATGGGCCAGGAAACACTC-3′	5′- GCCTGCAATGTCGTCCTTTG-3′
*MMP-13*	5′-AAGCACCCCAAAACACCAGA-3′	5′- ACATGAGGTCTCGGGATGGA-3′
*iNOS*	5′- CTTGGTGAGGGGACTGGACT-3′	5′- GGGGTTTTCTCCACGTTGTT-3′
*COX-2*	5′-TGGGAAGCTTTCTCCAACCT-3′	5′- GTGAAGTGCTGGGCAAAGAA-3′
*TNF-α*	5′- CATCCGTTCTCTACCCAGCC-3′	5′-TCCAGTGAGTTCCGAAAGCC-3′
*IL-6*	5′-TTCCTACCCCAACTTCCAAT-3′	5′- GCCAGTTCTTCGTCGAGAAC-3′
*IL-1β*	5′- TGGGATCCTCTCCAGTCAGG-3′	5′- ACACTGCCTTCCTGAAGCTC-3′
*GAPDH*	5′-TGGCCTCCAAGGAGTAAGAAAC-3′	5′-CAGCAACTGAGGGCCTCTCT-3′

**Table 2:** Effects of *Levilacillus brevis* KU15147 on organ weights in rats with MIA-induced osteoarthritis.

Weight (g/100 g body weight)	NC	MIA-induced arthritis			
		C	PC	15147-L	15147-H
Heart	0.34±0.01^a^	0.37±0.01^b^	0.35±0.01^ab^	0.36±0.01^ab^	0.36±0.01^ab^
Liver	3.01±0.05^a^	3.11±0.05^bc^	3.07±0.05^abc^	3.19±0.08^c^	2.92±0.05^a^
Spleen	0.23±0.00^a^	0.25±0.01^b^	0.24±0.01^ab^	0.24±0.01^ab^	0.24±0.01^ab^
Kidney	0.73±0.02^ns^	0.77±0.02	0.73±0.02	0.76±0.03	0.73±0.02

Data are expressed as g/100 g body weight and presented as the mean ± standard deviation (SD). Different letters indicate statistically significant differences among groups (*p* < 0.05).

NC: treatment without monosodium iodoacetate (MIA) injection; C: treatment with MIA injection; PC: treatment with MIA + indomethacin (3 mg/kg body weight/day); 15147-L: treatment with MIA + *L. brevis* KU15147 low-dose (1 × 10^8^ CFU/rat/day); 15147-H: treatment with MIA + *L. brevis* KU15147 (1 × 10^9^ CFU/rat/day).

**Table 3 T3:** Morphological analysis of cortical bone in rats with MIA-induced osteoarthritis using micro-CT.

	NC	MIA-induced arthritis			
		C	PC	15147-L	15147-H
BMD (g/cm^2^)	818.6±4.0^b^	794.7±8.1^a^	819.3±6.9^b^	805.8±4.8^ab^	818.2±5.4^b^
BV/TV	0.22±0.01^b^	0.18±0.01^a^	0.16±0.01^a^	0.15±0.00^a^	0.16±0.01^a^
Tb.N (1/mm)	1.28±0.02^b^	1.24±0.02^bc^	1.16±0.06^ab^	1.07±0.02^a^	1.13±0.06^ab^
Tb.Th (mm)	0.18±0.01^b^	0.14±0.01^a^	0.14±0.00^a^	0.14±0.00^a^	0.14±0.01^a^
Tb.Sp (mm)	0.60±0.02^a^	0.66±0.02^ab^	0.72±0.03^bc^	0.79±0.02^c^	0.75±0.05^bc^

Data are presented as the mean ± standard deviation (SD). Different letters indicate statistically significant differences among groups (*p* < 0.05).

BMD: bone mineral density; BV/TV: bone volume fraction; Tb, N: trabecular number; Tb.Th: trabecular thickness; Tb.Sp: trabecular separation.

NC: treatment without monosodium iodoacetate (MIA) injection; C: treatment with MIA injection; PC: treatment with MIA + indomethacin (3 mg/kg body weight/day); 15147-L: treatment with MIA + *L. brevis* KU15147 low-dose (1 × 10^8^ CFU/rat/day); 15147-H: treatment with MIA + *L. brevis* KU15147 (1 × 10^9^ CFU/rat/day).

**Table 4:** Effects of *Levilactobacillus brevis* KU15147 on knee joint thickness in rats with MIA-induced osteoarthritis.

Group	Day 7 (mm)	Day 10 (mm)	Day 13 (mm)
NC	20.0±0.3^a^	17.3±0.2^a^	17.2±0.2^a^
C	21.4±0.2^a^	18.8±0.4^b^	18.8±0.5^c^
PC	20.2±0.2^a^	18.1±0.2^ab^	17.8±0.2^bc^
15147-L	20.5±0.2^a^	17.8±0.2^ab^	18.0±0.2^ab^
15147-H	20.4±0.2^a^	18.0±0.3^a^	18.0±0.2^ab^

Data are presented as the mean ± standard deviation (SD). Different letters indicate statistically significant differences among groups (*p* < 0.05).

Changes in knee joint thickness were measured at 7, 10, and 13 days after MIA injection.

NC: treatment without monosodium iodoacetate (MIA) injection; C: treatment with MIA injection; PC: treatment with MIA + indomethacin (3 mg/kg body weight/day); 15147-L: treatment with MIA + *L. brevis* KU15147 low-dose (1 × 10^8^ CFU/rat/day); 15147-H, treatment with MIA + *L. brevis* KU15147 (1 × 10^9^ CFU/rat/day).

## References

[1] Súan Tang, Changqing Zhang, Win Min Oo, Kai Fu, May Arna Risberg, Sita M Bierma-Zeinstra, *et al*. 2025. Osteoarthritis. *Nat. Rev. Dis. Primers* **11:** 10. https://doi.org/10.1038/s41572-025-00594-6. 10.1038/s41572-025-00594-6 39948092

[2] Hunter DJ, Bierma-Zeinstra S (2019). Osteoarthritis. Lancet.

[3] Goldring MB, Goldring SR (2010). Articular cartilage and subchondral bone in the pathogenesis of osteoarthritis. Ann. N. Y. Acad. Sci..

[4] Kapoor M, Martel-Pelletier J, Lajeunesse D, Pelletier JP, Fahmi H (2011). Role of proinflammatory cytokines in osteoarthritis. Nat. Rev. Rheumatol..

[5] Batarfi WA, Yunus MHM, Hamid AA, Maarof M, Abdul Rani R (2025). Breaking down osteoarthritis: Exploring inflammatory and mechanical signaling pathways. Life (Basel).

[6] Michelacci YM, Baccarin RYA, Rodrigues NNP (2023). Chondrocyte homeostasis and differentiation: transcriptional control and signaling in healthy and osteoarthritic conditions. Life (Basel).

[7] Poole AR (2012). Osteoarthritis as a whole joint disease. HSS J..

[8] Wojdasiewicz P, Poniatowski LA, Szukiewicz D (2014). The role of inflammatory mediators in osteoarthritis. Mediators Inflamm..

[9] Mukherjee A, Das B (2024). The role of inflammatory mediators and matrix metalloproteinases (MMPs) in the progression of osteoarthritis. Biomater. Biosyst..

[10] Hu W, Chen Y (2021). MMP-13 and its role in osteoarthritis. Int. J. Mol. Sci..

[11] Coates-Park S, Rich JA, Stetler-Stevenson WG, Peeney D (2024). The TIMP protein family: diverse roles in pathophysiology. Am. J. Physiol. Cell Physiol..

[12] Manoj Arra, Gaurav Swarnkar, Ke Ke, Jesse E Otero, Jun Ying, Xin Duan, *et al*. 2020. LDHA-mediated ROS generation in chondrocytes is a potential therapeutic target for osteoarthritis. *Nat. Commun.* **11:** 3427. https://doi.org/10.1038/s41467-020-17242-0. 10.1038/s41467-020-17242-0 32647171 PMC7347613

[13] Fan F, Yang C, Piao E, Shi J, Zhang J (2024). Mechanisms of chondrocyte regulated cell death in osteoarthritis: Focus on ROS-triggered ferroptosis, parthanatos, and oxeiptosis. Biochem. Biophys. Res. Commun..

[14] Liu-Bryan R (2015). Inflammation and intracellular metabolism: New targets in OA. Osteoarthr. Cartil..

[15] Bao Z, Chen M, Li C, Shan Q, Wang Y, Yang W (2022). Monosodium iodoacetate-induced subchondral bone microstructure and inflammatory changes in an animal model of osteoarthritis. Open Life Sci..

[16] Pitcher T, Sousa-Valente J, Malcangio M (2016). The monoiodoacetate model of osteoarthritis pain in the mouse. J. Vis. Exp..

[17] da Costa BR, Reichenbach S, Keller N, Nartey L, Wandel S, Jüni P (2017). Effectiveness of non-steroidal anti-inflammatory drugs for the treatment of pain in knee and hip osteoarthritis: A network meta-analysis. Lancet.

[18] Bally M, Dendukuri N, Rich B, Nadeau L, Helin-Salmivaara A, Garbe E (2017). Risk of acute myocardial infarction with NSAIDs. BMJ.

[19] Plaza-Diaz J, Ruiz-Ojeda FJ, Gil-Campos M, Gil A (2019). Mechanisms of action of probiotics. Adv. Nutr..

[20] Hill C, Guarner F, Reid G, Gibson GR, Merenstein DJ, Pot B (2014). The ISAPP consensus statement on probiotics. Nat. Rev. Gastroenterol. Hepatol..

[21] Haddad BI, Lubbad MA, Mahmoud HA, Jrasat LR, Jilani IK, Ali Y (2025). Gut microbiota in osteoarthritis: Impact on disease progression and symptom management. A systematic review. J. Orthop. Rep..

[22] Chen Y, Zhang L, Li E, Zhang G, Hou Y (2021). Probiotic supplementation attenuates experimental osteoarthritis. Nutrients.

[23] Hyun JH, Yu HS, Woo IK, Lee GW, Lee NK, Paik HD (2023). Anti-inflammatory activities of *Levilactobacillus brevis* KU15147 in RAW 264.7 cells stimulated with lipopolysaccharide via attenuation of NF-κB, AP-1, and MAPK signaling pathways. Food Sci. Biotechnol..

[24] Kapoor M, Martel-Pelletier J, Lajeunesse D, Pelletier JP, Fahmi H (2017). Role of proinflammatory cytokines in the pathophysiology of osteoarthritis. Nat. Rev. Rheumatol..

[25] Lei M, Guo C, Wang D, Zhang C, Hua L (2023). Probiotics in osteoarthritis: a systematic review and meta-analysis. Front. Pharmacol..

[26] Woo IK, Hyun JH, Jang HJ, Lee NK, Paik HD (2025). Probiotic *Pediococcus acidilactici* strains exert anti-inflammatory effects by regulating intracellular signaling pathways in LPS-induced RAW 264.7 cells. Probiotics Antimicrob. Proteins.

[27] Attur M, Krasnokutsky-Samuels S, Samuels J, Abramson SB (2013). Prognostic biomarkers in osteoarthritis. Curr. Opin. Rheumatol..

[28] Song M, Kim WJ, Shim J, Song K (2025). *Latilactobacillus sakei* LB-P12 ameliorates osteoarthritis by reducing cartilage degradation and inflammation via regulation of NF-κB/HIF-2α pathway. J. Microbiol. Biotechnol..

[29] Goldring MB, Otero M (2011). Inflammation in osteoarthritis. Curr. Opin. Rheumatol..

[30] Brew K, Nagase H (2010). The tissue inhibitors of metalloproteinases (TIMPs): an ancient family with structural and functional diversity. Biochim. Biophys. Acta Mol. Cell Res..

[31] Zhen G, Cao X (2014). Targeting TGF-β signaling in subchondral bone and articular cartilage homeostasis. Trends Pharmacol. Sci..

[32] Dudaric L, Dumic-Cule I, Divjak E, Cengic T, Brkljacic B, Ivanac G (2023). Bone remodeling in osteoarthritis-biological and radiological aspects. Medicina (Kaunas).

[33] Choi MC, Jo J, Park J, Kang HK, Park Y (2019). NF-κB signaling pathways in osteoarthritic cartilage destruction. Cells.

[34] Huang Z, Kraus VB (2016). Does lipopolysaccharide-mediated inflammation have a role in OA?. Nat. Rev. Rheumatol..

[35] Ron Gilat, Allen A Yazdi, Alexander C Weissman, Kaitlyn M Joyce, Fatima A Bouftas, *et al*. 2025. The gut microbiome and the joint microbiome show alternations in patients with knee osteoarthritis versus controls: A systematic review. *Arthroscopy* **41:** 1226-1238. https://doi.org/10.1016/j.arthro.2024.05.010. 10.1016/j.arthro.2024.05.010 38797504

